# Label-free X-ray estimation of brain amyloid burden

**DOI:** 10.1038/s41598-020-77554-5

**Published:** 2020-11-25

**Authors:** Eshan Dahal, Bahaa Ghammraoui, Meijun Ye, J. Carson Smith, Aldo Badano

**Affiliations:** 1 Division of Imaging, Diagnostics and Software Reliability, Office of Science and Engineering Laboratories, Center for Devices and Radiological Health, Food and Drug Administration, Silver Spring, MD 20993 USA; 2 Fischell Department of Bioengineering, University of Maryland, College Park, MD 20742 USA; 3 Division of Biomedical Physics, Office of Science and Engineering Laboratories, Center for Devices and Radiological Health, Food and Drug Administration, Silver Spring, MD 20993 USA; 4 School of Public Health, University of Maryland, College Park, MD 20742 USA

**Keywords:** Biological techniques, Alzheimer's disease, Biomedical engineering

## Abstract

Amyloid plaque deposits in the brain are indicative of Alzheimer’s and other diseases. Measurements of brain amyloid burden in small animals require laborious post-mortem histological analysis or resource-intensive, contrast-enhanced imaging techniques. We describe a label-free method based on spectral small-angle X-ray scattering with a polychromatic beam for in vivo estimation of brain amyloid burden. Our findings comparing 5XFAD versus wild-type mice correlate well with histology, showing promise for a fast and practical in vivo technique.

## Introduction

The presence of $\beta$-sheet rich amyloid plaques in the brain has been associated with a wide range of neurological diseases including Alzheimer’s and Parkinson’s^1,2^. Amyloid plaques in Alzheimer’s disease (AD) are formed by aggregation of amyloid-$\beta$ (A$\beta$) peptides into fibrils and their extracellular deposition in the brain^1^. Measurements of amyloid burden require laborious post-mortem histological analysis or resource-intensive contrast-enhanced imaging techniques. As A$\beta$ plaques accumulate in the brain, in vivo estimation of amyloid burden is necessary for understanding AD pathogenesis and progression, and for designing diagnostic and therapeutic approaches. Positron emission tomography (PET) uses radiolabeled compounds that bind to amyloid fibrils with moderate specificity^3^. While this method is clinically employed, its use has been limited due to cost and availability of PET scanners and by the need to inject a radioactive substance in humans. In small-animal preclinical studies, amyloid PET has provided unconvincing results even for AD mouse models with high amyloid load in part due to non-specific binding^4,5^. More recently, magnetic resonance imaging (MRI) has shown potential for quantifying amyloid burden in vivo in various AD mouse models. However, MRI techniques depend on administering an exogenous contrast agent typically based on gadolinium compounds^6^. The need for readily assessing amyloid burden in vivo without using contrast agents has not yet been resolved. We demonstrate a label-free, fast and non-invasive method based on spectral small-angle X-ray scattering (sSAXS) to estimate amyloid burden by studying wild-type and 5XFAD mice with a prototype system and correlating the estimated amyloid burden with histology.

Small-angle X-ray scattering (SAXS) is a well-established technique for identifying molecular structures based on their scattering patterns^7,8^. SAXS is limited to the study of thin samples (mm scale) with the use of low-energy monochromatic x rays. In sSAXS, we improve system efficiency by simultaneously collecting SAXS data in angle- and energy-dispersive modes using a polychromatic X-ray beam and a 2D spectroscopic detector. In this approach, elastically scattered x rays at angles below $10^{\circ }$ are acquired by the detector with no energy filtering. For each detected quanta, the detector records the energy and location information into an three-dimensional image output. This approach overcomes the sample thickness limit of traditional SAXS and allows the exploration of in vivo application with higher energies utilizing all available scattering flux in the energy range of interest. Using Monte Carlo simulations, we have shown that the energy range can be selected to maximize elastic scattering per deposited energy in thick samples^9^. In another study, we have experimentally demonstrated the capability for identifying embedded targets in up to 5-cm-thick objects^10^ and in wild-type excised mouse heads^11^ with an energy range from 30 to 45 keV.

## Results and discussion

**Figure 1:**
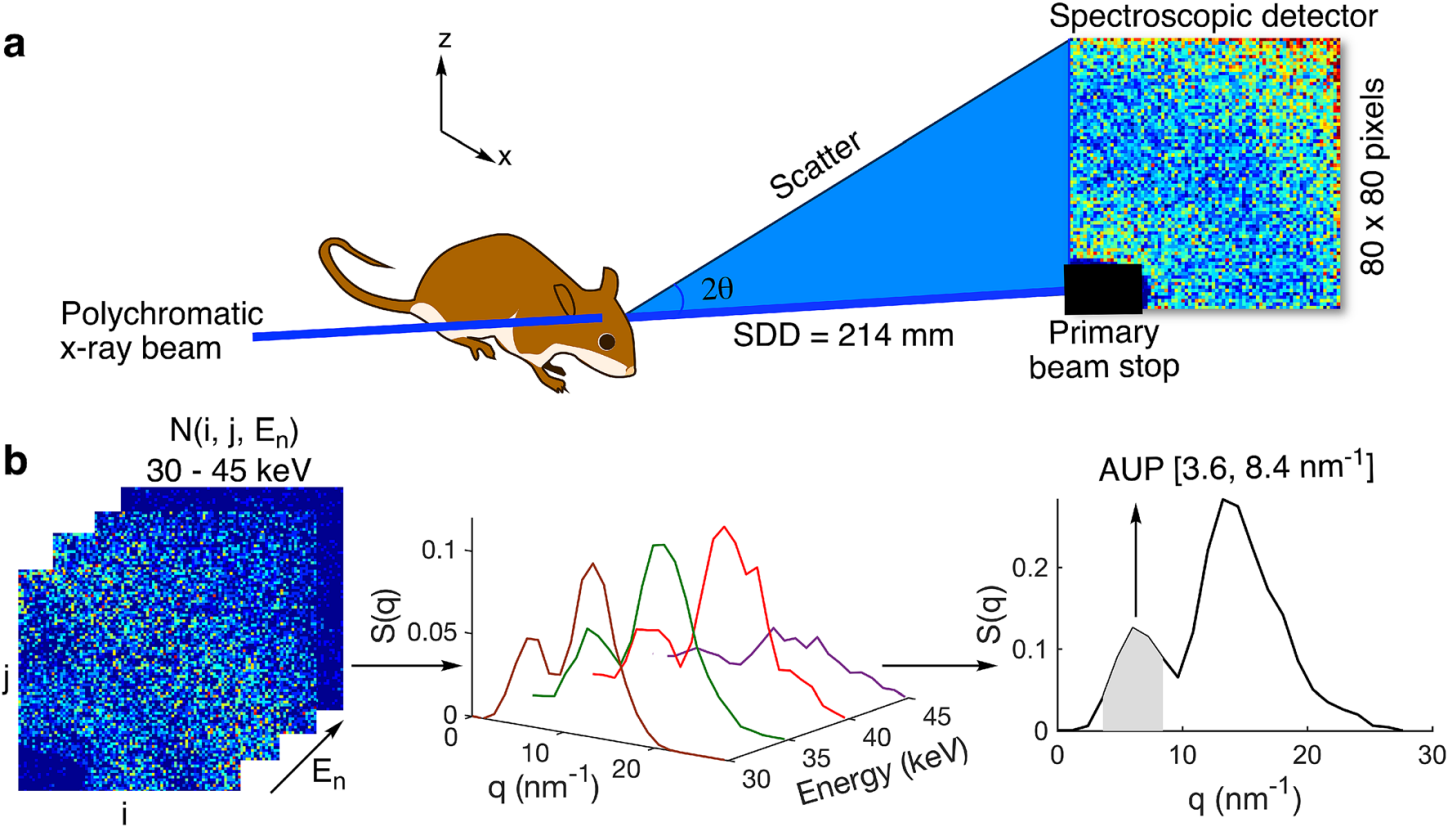
Schematic of the experimental sSAXS setup and data processing steps. (**a**) The mouse head is irradiated at select locations with a collimated beam of polychromatic X rays of 1 mm diameter. A 2D spectroscopic detector (CdTe) is used to collect SAXS data simultaneously in angle- and energy-dispersive modes for each location. The sample to detector distance (SDD) was 214 mm (**b**) sSAXS data processing steps in 30–45 keV energy range are illustrated using an amyloid model from BSA with two Bragg peaks at 6.04 and $13.24\,{\hbox {nm}}^{-1}$. The 2D detector data (left) showing counts in each pixel per energy bin is converted to scattering cross section, *S*(*q*), per energy bin (middle) after applying a transmission correction. The *S*(*q*) is then summed ($\Delta \hbox {q} = 1.2 \, {\hbox {nm}}^{-1}$) from 30 to 45 keV to combine scattering information in all energy bins and to calculate the area under the peak (AUP) from 3.6 to $8.4 \, {\hbox {nm}}^{-1}$ (right).

Figure 1a shows a schematic of the experimental sSAXS setup. A dual-pinhole collimated beam of polychromatic X rays is directed at the mouse head in selected locations in a sagittal plane between eye and ear. The X-ray beam path is normal to the sagittal plane. The sSAXS data processing steps are illustrated using data collected for an amyloid model in Fig. 1b. First, the detector data in the energy range from 30 to 45 keV is transmission corrected and converted to scattering cross section, *S*(*q*), per energy bin. The *S*(*q*) is then summed for all energy bins. Using these processing steps (see Methods section for details), we see two characteristic peaks for the amyloid model at 6.04 and $13.24\,{\hbox {nm}}^{-1}$ due to its cross-$\beta$ structure^2,12^. X-ray diffraction methods have identified the same two peaks when characterizing amyloid-rich thin tissue sections of mouse and human brain^2,13^. Since the signal increases with amyloid plaque density^11^, we used the area under the peak (AUP) from 3.6 to $8.4\,{\hbox {nm}}^{-1}$ to estimate the amyloid burden. We chose not to use the broad peak at $13.24\,{\hbox {nm}}^{-1}$ because of significant overlap with the scattering signal from the lipid content of brain tissues^14^.

**Figure 2:**
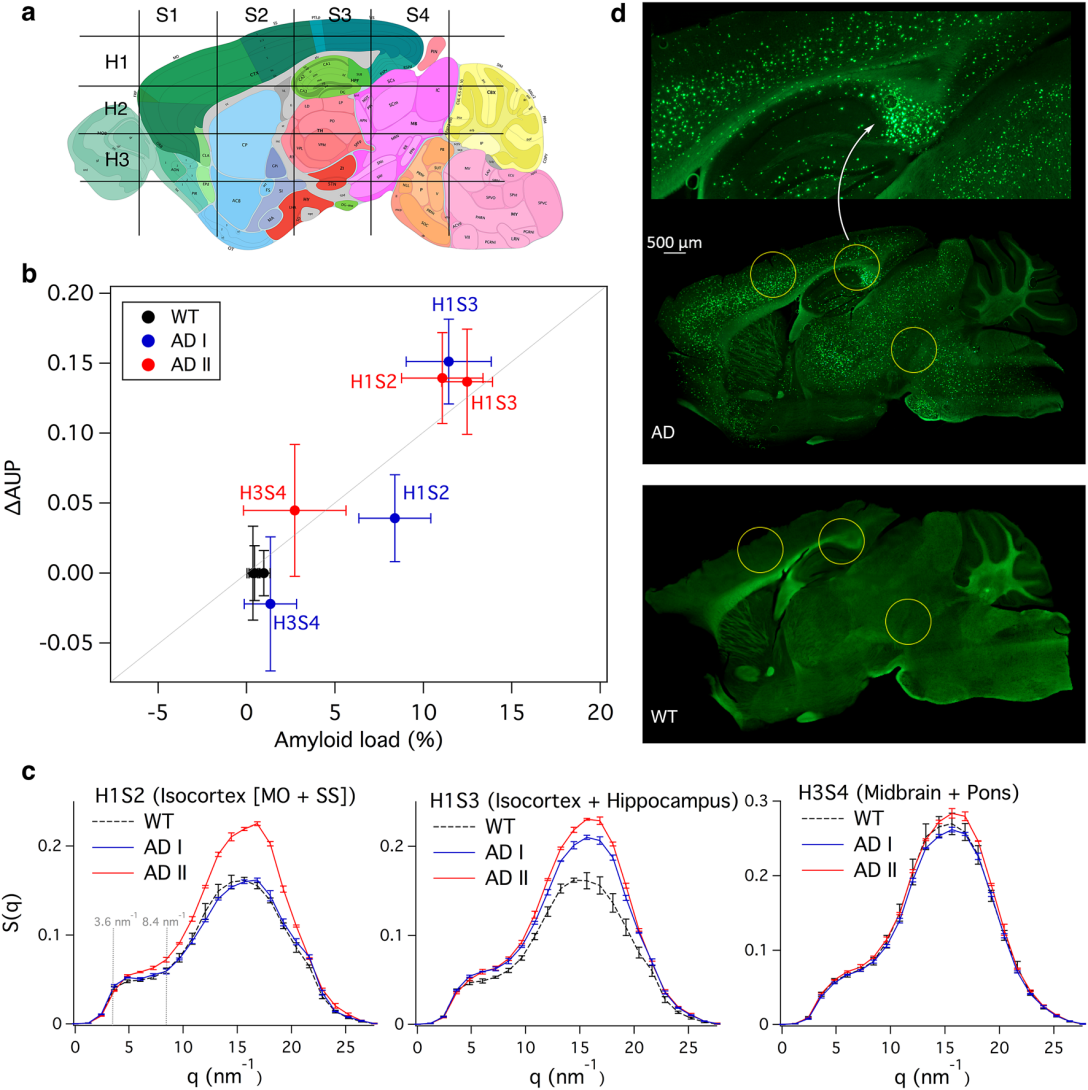
Amyloid burden estimations in intact heads corresponding to 5XFAD mice and WT controls. (**a**) Approximate studied locations in the mouse brain in comparison to an anatomic reference image from the Allen Mouse Brain Atlas containing major brain regions in a sagittal plane^25^. (**b**) Correlation between the $\Delta$AUP calculated from the sSAXS method and the amyloid load estimates derived from histology for three locations (Pearson $r = 0.94, P < 0.001$). (**c**) Corresponding S(q) of AD and WT mice used for $\Delta$AUP calculation after studying their heads with an intact skull in each location for 300 s. Error bars represent ± standard deviation from three measurements for each location. (**d**) Representative histology images of AD and WT brain slices using Thioflavin S. The zoomed image (top) is focused on the hippocampus of AD brain slice.

To non-invasively locate the selected region-of-interest (ROI) in the mouse heads, we acquire a scout scan for a set of AD and WT mouse heads between eye and ear (sagittal plane, $\Delta \hbox {x}=2\,\hbox {mm}$) at different axial heights ($\Delta \hbox {z}=1\,\hbox {mm}$) with a 0.5 mm X-ray beam. The mouse heads were then cut into halves to find the approximate ROI locations using an anatomic atlas as visual reference (Fig. 2a). We chose three ROIs (H1S2, H1S3, H3S4) for a detailed study with a 1-mm X-ray beam based on the scout scan (Supplementary Fig. 2). H1S2 and H1S3 showed a strong signal from amyloid plaques, whereas H3S4 showed weak or no signal. As shown in Fig. 2a, H1S2 corresponds to the isocortex’s somatomotor and somatosensory regions, H1S3 corresponds to the isocortex’s visual, posterior parietal association and somatosensory regions as well as to the hippocampus (hippocampal and retrohippocampal regions), and H3S4 to midbrain and pons regions. We chose a 1-mm X-ray beam to improve signal-to-noise ratio while curtailing smearing (Supplementary Fig. 3). $\Delta$AUP (represented as fraction) relative to the WT mice was used to estimate amyloid burden in each location (see Methods section). For comparison, histological analysis was performed to estimate amyloid load (%) after completing the sSAXS study. First, the skull was removed and the brain was sliced into 50-$\upmu \hbox {m}$ sagittal sections. Two slices from each hemisphere of the brain at lateral distances of 1 and 2 mm were stained with 0.1% Thioflavin-S solution. The average of four brain slices ± standard deviation was used to report amyloid load for each location (see Methods).

We found a strong correlation (Pearson $r = 0.94, P<0.001$) between the $\Delta$AUP calculated with our sSAXS method and the amyloid load estimates derived from histology (Fig. 2b). Tabulated data are provided in Supplementary Table 1. Figure 2c shows *S*(*q*) of AD and WT mice used for $\Delta$AUP calculation, where the AUP is taken from 3.6 to $8.4 \, {\hbox {nm}}^{-1}$. Consistent with histology, the maximum $\Delta$AUP ($0.15 \pm 0.03$) was observed in H1S3 of the first 5XFAD mouse (AD I) corresponding to the isocortex and hippocampus regions. $\Delta$AUP for the second 5XFAD mouse (AD II) in H1S3 and H1S2 also showed high amyloid burden. Typical of 11 months old 5XFAD mice^15^, amyloid plaques were concentrated in the subiculum corresponding to H1S3 and distributed in the inner layers of the cortex as seen in H1S2 (Fig. 2d and Supplementary Fig. 4). We found $\Delta$AUP in H1S2 of AD I to be slightly lower than that measured by histology likely due to a mismatch in the studied location within the isocortex while placing the mouse head in the X-ray beam. We observed a $\Delta$AUP of $-\,0.02 \pm -\,0.05$ in H3S4 of AD I corresponding to the midbrain and pons regions. A zero or negative $\Delta$AUP indicates no significant presence of amyloid plaques. In H3S4 of AD I, the histological amyloid load was $1.4 \pm 1.5\%$. We saw a positive $\Delta$AUP value on average for H3S4 of AD II. The average histological amyloid load in this case was about twice that seen in AD I. Our sSAXS method was sensitive enough to detect a $2.7 \pm 2.9\%$ amyloid load reported by histology. The primary source of variability in AUP is due to location uncertainty while repositioning the mouse head for three consecutive measurements in the 1-mm X-ray beam. The variability contributed by the instrument alone is less than 10%^10^. Our sSAXS method can be improved by decreasing location uncertainty. A two-dimensional scout X-ray image taken with the same detector in transmission mode could allow more accurate target localization prior to the start of the sSAXS acquisition. A three-dimensional scout image using a fast (under-sampled) accessory computed tomography setup could provide additional location accuracy.

Radiation levels might limit in vivo applications if long exposure times are required to study multiple brain locations. Exposure time can be reduced by increasing photon counts in the energy range of interest (30–45 keV) through various system optimizations. In addition, X-rays with energy below 30 keV can be filtered out from the beam before irradiating the sample to reduce absorbed dose. It is worth emphasizing that amyloid plaques in the brain are present in a wide range of diseases and conditions^1,16,17^, including prion^18^, Parkinson’s^2^, and traumatic brain injury^19^. Therefore, we can quickly diversify the application of our method in the future by using the same detection mechanism focusing on the scattering signal from $\beta$-sheet structures that locally increases in amyloid plaques^20^. In this work, we used the 5XFAD model that rapidly develops amyloid pathology in order to detect and quantify amyloid plaques only^15^. Despite promising results, a limitation of our study is the small sample size. However, we performed a total of 36 measurements with three repeats with head repositioning for each ROIs. These measurements were done after scanning an initial set of separate AD and WT mice to select and locate the three ROIs. Future work is needed to estimate amyloid burden in an age-dependent manner with a larger sample size in both ex vivo experiments and in live animals. It is also worthwhile to use other AD mouse models to test our method with and without the presence of tau tangles^21^. Since the energy range of X-rays can be tuned to probe thick samples, there is also an opportunity to use sSAXS to identify amyloid plaques in the human head in the energy range between 50 and 120 keV, as supported by recent Monte Carlo simulation studies^22–24^. Therefore, the overall outlook of the sSAXS method that we have demonstrated here by studying a transgenic mouse model of AD is promising.

## Conclusion

We studied 5XFAD mice and demonstrated the capability of a novel sSAXS method to estimate brain amyloid burden in mice without contrast agents and in a non-invasive manner. The sSAXS method enables the in vivo estimation of amyloid burden by integrating a polychromatic X-ray source with a 2D spectroscopic detector. The method is relatively fast (a few minutes) and convenient for the analysis of amyloid burden in small animals within a preclinical setting.

## Methods

### Amyloid model

We prepared an amyloid model using bovine serum albumin (BSA) powder (Sigma-Aldrich, St. Louis, MO) with cross $\beta$-sheet structures similar to amyloid plaques seen in AD patients^12^. A 6.4 mm diameter plastic syringe was filled with BSA powder to a packing density of $758 \pm 54 \, {\hbox {mg/cm}}^{3}$. The amyloid model was used to illustrate the data analysis process and show the scattering signal characteristic of $\beta$-sheet rich structures.

### Animals

All animal experimental procedures and protocols were approved by the Indiana University School of Medicine Institutional Animal Care and Use Committee (IACUC) and performed in accordance with US National Institutes of Health and AAALAC International guidelines and regulations on animal care. The 5XFAD mouse line contains the human amyloid precursor protein and presenilin transgenes with five mutations associated with AD^15^. 5XFAD mice exhibit severe A$\beta$ deposition at an early age. A$\beta$ continues to accumulate until 10–14 months of age. 5XFAD mice (n = 3) and nontransgenic wildtype (WT) mice (n=3) on the same background were aged 11 months. Animals were housed at Indiana University School of Medicine Laboratory Animal Resource Center under a 12 h light/dark cycle. Animals had access to food and water *ad libitum*. Mice were transcardially perfused with 1$\times$ phosphate buffered saline and formaldehyde. Heads were removed from the body and shipped to our lab to be used in the study (Supplementary Fig. 1). Two sets of AD and WT mouse heads were used for the sSAXS study followed by the histological analysis. A separate set of AD and WT mouse heads were used for initial testing in the sSAXS study to scan and locate the region of interest.

### Experimental sSAXS setup

We built a sSAXS system by assembling a polychromatic X-ray source (tungsten anode, MXR-160/22, COMET) and a 2D spectroscopic detector made from 1-mm thick cadmium telluride (HEXITEC, Quantum Detectors Ltd, Oxfordshire, UK). The system is described in detail elsewhere^10^. Briefly, polychromatic x rays were generated using a 50-kVp tube voltage and 2-mA current. The beam is collimated to irradiate the mouse head using two lead pinholes of 2.5 and 1.0 mm diameter respectively. The two pinholes were 160 mm apart and the sample to detector distance (SDD) was 214 mm. The detector ($80 \times 80$ 250-$\upmu \hbox {m}$ pixels) recorded scattered photons with energies between 6 and 50 keV using a bias voltage of − 400 V, a 5-keV energy threshold and 1-keV energy bins. The image acquisition time was 300 s. A beamstop in front of the detector was used to block the primary rays to avoid pulse pile-up while recording the scattered photons. An energy-dependent transmission correction factor was calculated using spectroscopic measurements of the primary beam with and without the sample present, without the beam stop and using a 0.2-mA tube current, and a 27-keV energy threshold^10^.

### Data analysis

Similarly to traditional SAXS, sSAXS analyzes scattering data in terms of momentum transfer *q*^10^. The scattered photons recorded at each pixel have a known scattering angle ($2\theta$) and energy (*E*). The momentum transfer is given by

1 $$\begin{aligned} q = \frac{4\pi E \sin \theta }{hc} , \end{aligned}$$

where $hc = 1.24\,\hbox {keV\,nm}$. The q range analyzed in our sSAXS setup was from 1.3 to $28 \, {\hbox {nm}}^{-1}$ with *E* from 30 to 45 keV. Let $N_{s}(i,j,E)$ be the detector data corresponding to scattered photon counts in each pixel within an energy bin, where (i, j) denotes the pixel index. If *T* is the sample thickness along the X-ray path, $N_{s}(i,j,E)$ can be written as

2 $$\begin{aligned} {N_{s}(i,j,E) = N_{t}(i,j,E)\int _{0}^{T}S(x,\theta _{ij},E)dx} , \end{aligned}$$

where $N_{t}(i,j,E)$ is the photon counts of primary (unscattered) quanta after transmitting through the sample and $S(x,\theta _{ij},E)$ is the angular and energy scattering cross-section. $N_{s}(i,j,E)$ was divided by $N_{t}(i,j,E)$ to account for energy-dependent transmission and detector response^10,26^. Equation 1 represents the scattering cross-section data as a function of *q* per energy bin. Finally, *S*(*q*) was summed for all energy bins (30–45 keV). The bin size in *q* was $1.2 \, {\hbox {nm}}^{-1}$. We then calculate the area under the peak (AUP) from 3.6 to $8.4 \, {\hbox {nm}}^{-1}$. $\Delta$AUP represents the amyloid burden per location (*i*) calculated as

3 $$\begin{aligned} \Delta {AUP} = \frac{AUP_i-{\overline{AUP}}_{WT}}{{\overline{AUP}}_{WT}}, \end{aligned}$$

where $AUP_i$ is the AUP in each location under investigation and ${\overline{AUP}}_{WT}$ is the mean of all ROIs in the WT mouse (n=2). All locations in AD and WT mice were location-matched for comparison. $\Delta$AUP represents a relative measurement of amyloid burden compared to WT measurements. The theoretical minimum value for $\Delta$AUP is 0 and corresponds to a level seen in WT indicative of non-disease. However, its maximum value can be greater than 1. In order to scale $\Delta$AUP from 0 to 1, one could use an age-dependent mice study with appropriate sample size to find minimum and maximum population-based AUP values, similarly to the recently proposed Centiloid metric for standardizing amyloid PET data^27^.

### Histology

Histological analysis was performed using a Thioflavin-S staining protocol^28^. The skull was removed from the mouse head to slice the brain into $50\,\upmu \hbox {m}$ sagittal sections using a vibrating microtome. Two slices from each hemisphere of the brain (total four slices per brain) at lateral distances of 1 and 2 mm were stained with 0.1% Thioflavin-S solution. The slices were then mounted onto glass slides and imaged using a fluorescence microscope (Axio Observer, Zeiss). ImageJ software (https://imagej.nih.gov/ij/) was used to calculate the amyloid load as percent area (percent number of pixels in the image) covered by the plaques in the ROI^29^. Three locations were selected for analysis based on the sSAXS study. ROI was defined to be a 1 mm circle matching the size of the X-ray beam. The Allen Mouse Brain Atlas^25^ was used to identify the brain regions. Sections in the brain sagittal plane were divided based on the scanning step size from our sSAXS measurements with $\Delta \hbox {x} = 2 \, \hbox {mm}$ and $\Delta \hbox {z} = 1 \, \hbox {mm}$. The mouse eye and ear were used as a reference to determine the start and endpoint for scanning. A set of AD and WT mouse heads were also cut into half to trace the X-ray path visually and confirm the approximate irradiated location with respect to an anatomic atlas^25^. Images were background subtracted to match average pixel values of WT and AD brain slices in regions without amyloid plaques, and thresholding was applied to highlight any Thioflavin-S positive plaques. The percent area of plaques in the circular ROI was calculated. For each 5XFAD mouse, the average of four slices was reported as the amyloid load for each location.

## Supplementary information

Supplementary information.
