# Supplementary material for: Label-free X-ray estimation of brain amyloid burden
Source: Sci Rep. 2020 Nov 25;10:20505. doi: 10.1038/s41598-020-77554-5 (PMC7689528; doi:10.1038/s41598-020-77554-5)
Supplement: Supplementary file 1 — Supplementary information. [file 41598_2020_77554_MOESM1_ESM.pdf]

# Supplementary Information for "Label-free x-ray estimation of brain amyloid burden"

**Eshan Dahal[1,2], Bahaa Ghammraoui[1], Meijun Ye[3], J. Carson Smith[4], & Aldo Badano[1,2*]**

[1]Division of Imaging, Diagnostics, and Software Reliability, Office of Science and Engineering Laboratories, Center for Devices and Radiological Health, Food and Drug Administration, Silver Spring, MD 20993, USA

[2]Fischell Department of Bioengineering, University of Maryland, College Park, MD20742, USA

[3]Division of Biomedical Physics, Office of Science and Engineering Laboratories, Center for Devices and Radiological Health, Food and Drug Administration, Silver Spring, MD 20993, USA

[4]School of Public Health, University of Maryland, College Park, MD 20742, USA

*E-mail:aldo.badano@fda.hhs.gov

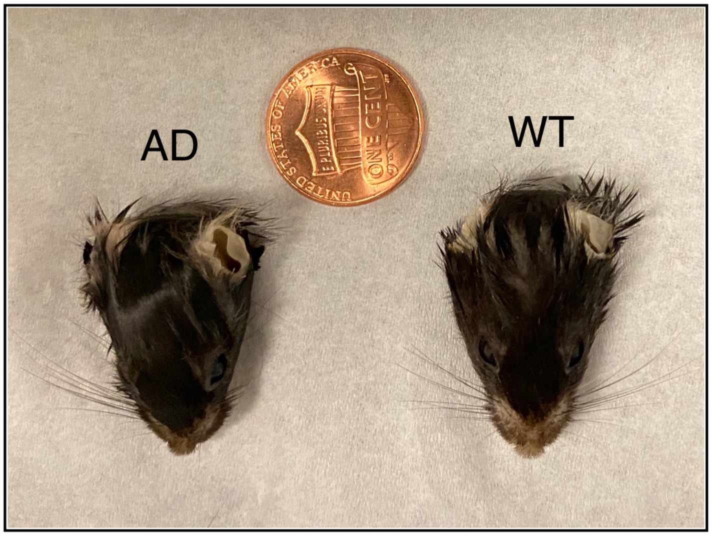


**Supplementary Figure 1.** AD and WT mouse head samples used in the sSAXS study.

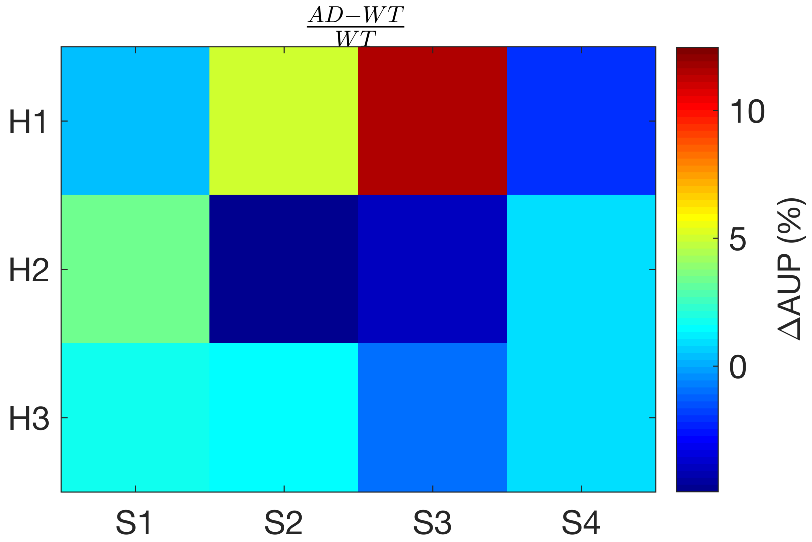


**Supplementary Figure 2.** Initial AD and WT mouse head scanning results with a 0.5 mm polychromatic x-ray beam. Each mouse head was scanned between eye and ear (S1-S4, sagittal plane, $\Delta x = 2$ mm) at different heights (H1-H3, $\Delta z = 1$ mm).

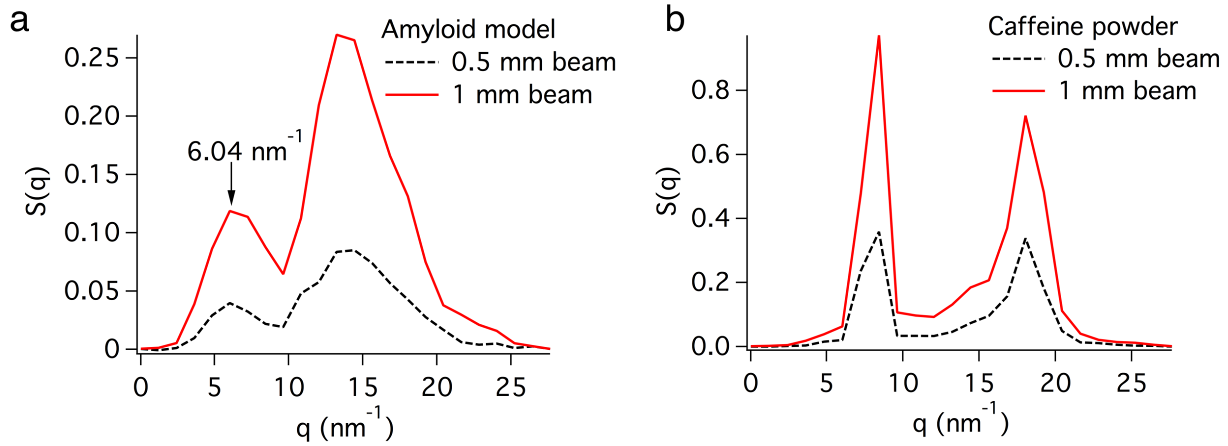


**Supplementary Figure 3.** S(q) of amyloid model (a) and caffeine (b) using 1 mm vs.0.5 mm x-ray beam spot size.

| | H1S2 (isocortex [MO + SS]) | | H1S3 (isocortex + hippocampus) | | H3S4 (midbrain + pons) | |
|---|---|---|---|---|---|---|
| | ΔAUP | Amyloid load (%) | ΔAUP | Amyloid load (%) | ΔAUP | Amyloid load (%) |
| WT | 0.00 ± 0.02 | 0.5 ± 0.3 | 0.00 ± 0.02 | 0.9 ± 0.4 | 0.00 ± 0.03 | 0.4 ± 0.3 |
| AD I | 0.04 ± 0.03 | 8.4 ± 2.0 | 0.15 ± 0.03 | 11.4 ± 2.4 | -0.02 ± -0.05 | 1.4 ± 1.5 |
| AD II | 0.14 ± 0.03 | 11.1 ± 2.3 | 0.14 ± 0.04 | 12.5 ± 1.4 | 0.04 ± 0.05 | 2.7 ± 2.9 |

**Supplementary Table 1.** Tabulated data of ΔAUP (fraction) and amyloid load (%).

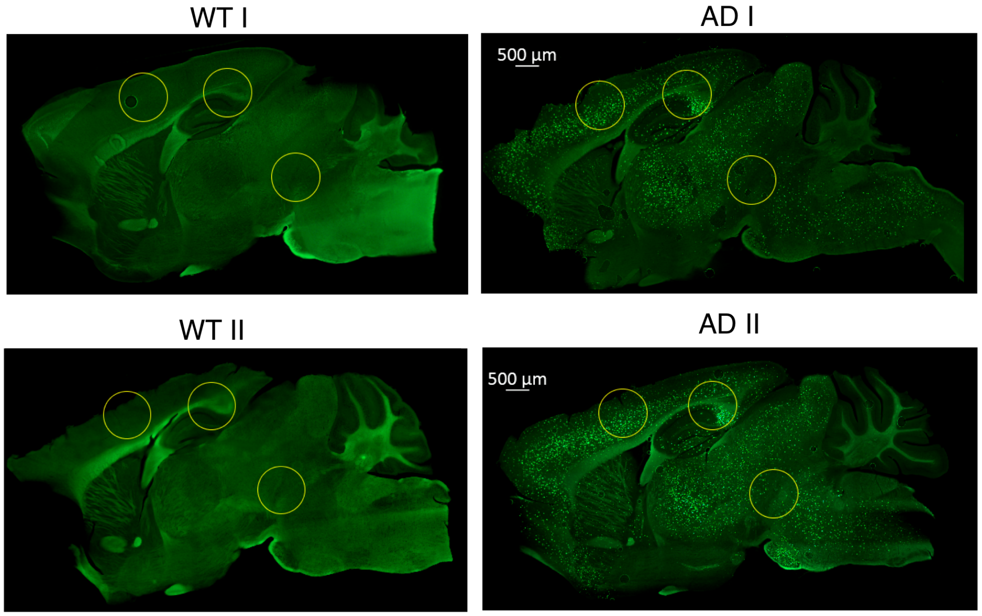


**Supplementary Figure 4.** Representative histology images of each mouse stained with Thioflavin S. Yellow circle shows ROI.
